# Leptin Levels and Nutritional Status of Indigenous Tepehuán and Mestizo Subjects in Durango, Mexico

**DOI:** 10.1155/2014/974503

**Published:** 2014-04-14

**Authors:** Dealmy Delgadillo Guzmán, Laurence Annie Marchat Marchau, José L. Reyes, Verónica Loera Castañeda, Martha Sosa Macías, Jessica García Vivas, Ismael Lares Asseff

**Affiliations:** ^1^Centro Interdisciplinario de Investigación para el Desarrollo Regional IPN, Durango, Sigma No. 119, Col. 20 de Noviembre II, 34220 Durango, DGO, Mexico; ^2^Escuela Nacional de Medicina y Homeopatía IPN Guillermo Massieu Helguera, No. 239, Fraccionamiento “La Escalera”, Ticoman, 07320 Mexico City, DF, Mexico; ^3^Centro de Investigación y de Estudios Avanzados del IPN Avenida Instituto Politécnico Nacional No. 2508, San Pedro Zacatenco, Gustavo A. Madero, 07360 Mexico City, DF, Mexico; ^4^Instituto Politécnico Nacional, CIIDIR, Durango, Mexico

## Abstract

The aim of this study was to assess differences in nutritional status and their association with circulating leptin levels in the indigenous Tepehuán people of Mezquital Durango and Mestizo populations of Durango City, Mexico. A group of 128 volunteers aged 18 through 59 years were recruited for the study: 60 indigenous Tepehuán from Mezquital and 68 Mestizo individuals from Durango City. The classification of nutritional status was through body mass index (BMI). Clinical evaluations, including anthropometry and lipid profiles, were performed to ascertain the health of the participants. Circulating leptin levels were determined in blood samples after at 08 hours of fasting. The healthy subjects were classified according to BMI: 32 Tepehuán and 30 Mestizo subjects were of normal weight (NW), and 28 Tepehuán and 38 Mestizo subjects were overweight or obese (OW/O). Both NW and OW/O Tepehuán subjects showed lower leptin concentrations than the comparable Mestizo subjects. Statistical analysis showed a negative Pearson's correlation (*r* = −0.5; *P* < 0.05) between BMI and leptin levels in NW Tepehuán subjects, but no significant correlation was found in other groups. The differences found in Tepehuán compared with Mestizo subjects might be explained by poor nutritional status, which leads to scarce adipose tissue and low levels of leptin synthesis. Leptin concentration and its relationship to BMI are associated with ethnicity.

## 1. Introduction

Leptin, a hormone mainly produced by adipose tissue, regulates energy homeostasis and has reproductive, neuroendocrine, immune, and metabolic functions [1]. Circulating leptin levels vary according to conditions such as nutritional status [1]. Thus, hyperleptinemia is an essential feature of human obesity. Diabetes does not influenceleptin secretion* per se* in lean and obese subjects. Independent of adiposity, leptin levels are higher in women than in men. Despite the strong correlation between body fat and leptin levels, there is great heterogeneity in leptin levels at any given index of body fat [1].

Isidori et al. [2] showed that in adults of different body weights, serum leptin gradually declines with age; this pattern is stronger in women than in men but it is independent of BMI and other age-related endocrine changes. Daniel et al. [3] investigated the effect of feeding or fat fasting on 24 h leptin profiles in thin ewes; they found that leptin plasma concentrations are episodic in nature and are influenced by nutritional status and fat thickness over the ribs but display no circadian variation.

Thomas et al. [4] found that fat mass, lean mass, and insulin levels are major determinants of serum leptin levels in adults. Moreover, after adjusting for these variables, the bioavailability of estrogens also explains a significant proportion of the variation in leptin levels in postmenopausal women on hormone-replacement therapy (HRT). Furthermore, Di Carlo et al. [5] noted that HRT after menopause results in unmodified, increased, or decreased leptin levels. Interestingly, circulating leptin levels also depend on ethnicity. Bribiesca [6] reports single-sample serum leptin levels in healthy Ache Amerindian males (*n* = 21; 32.8 ± 3.4 yrs) and females (*n* = 12; 31.3 ± 4.3 yrs) in eastern Paraguay. Leptin concentrations were much lower in the Ache subjects than in industrialized populations, although significant sexual dimorphism was evident (female, 5.64 ± 0.91 ng/mL versus male, 1.13 ± 0.08 ng/mL; *P* < 0.0001). Lilja et al. [7] found that Asian Indians have higher leptin levels and higher leptin: BMI and leptin: waist ratios than Creole and European individuals. Leptin has high intraindividual stability, and seasonal variation does not appear to explain the ethnic differences observed. AL-Harithy [8] reported gender-specific and age-dependent gender-specific differences in leptin concentrations in healthy Saudi individuals; however, unknown variables may also influence leptin levels in Saudi women and men. Waisberg et al. [9] suggested that the higher prevalence of obesity in African compared with Indian populations may be related to lower leptin levels; ethnic differences in the prevalence of metabolic disorders cannot be explained by differences in adipokine levels but may be related to higher visceral adiposity in the Indian group.

There has been no previous investigation of leptin levels in the indigenous groups of Mexico. We performed a comparative evaluation of the relationship between circulating leptin concentration and nutritional status in Tepehuán Amerindians, a native group that has inhabited northern Mexico since the second half of the 16th century [10], and Mestizo Mexicans.

## 2. Materials and Methods

### 2.1. Subjects and Ethical Aspects

The protocol was approved by the Ethical and Research Committees of Hospital General de Durango, SSA, Mexico. Male Tepehuán and Mestizo subjects (18 to 59 years old) from Durango State, Mexico, were recruited to the study. All participants were informed in their native language about the nature and purpose of the study and gave their consent to participate in the study by signing the informed-consent document. Clinical history and anthropometric measures (body weight in kg, height in m, median blood pressure (BP) in mm Hg) were determined for all participants. At each clinical examination, height, weight, and BP were measured using standardized procedures. Weight was measured using an electronic scale with participants wearing indoor clothing and no shoes. Height was measured under the same conditions to the nearest 0.5 cm using a wall-mounted stadiometer; blood pressure was taken once from each arm in participants who had been lying down for 10 min. The mean of those 2 measurements was used for analyses. If systolic BP (SBP) was >160 mm Hg and/or diastolic BP (DBP) was >100 mm Hg, pressures were remeasured after a second rest period of 10 min and the lowest value was retained. During the medical interview, two samples of 10 mL of venous blood were obtained from the left cubital vein after 12 hours of fasting. Both samples were collected in heparinized polypropylene tubes, mixed by inversion, and centrifuged at 3,000 rpm and 4°C for 10 min. The resulting plasma samples were aliquoted and stored at −70°C until analysis.

### 2.2. Assessment of Nutritional Status

Body mass index (BMI) is currently considered as a standard method for assessing nutritional status [11, 12]. BMI is a simple index of weight-for-height that is commonly used to classify underweight, overweight, and obesity in adults [13]. It is defined as the weight in kilograms divided by the square of the height in meters (kg/m^2^). BMI values are age-independent and the same for both sexes. The individuals were classified according to the criteria established by the FAO/WHO [13] as follows: BMI <18.5 kg/m^2^: underweight; 18.5 kg/m^2^ ≤ BMI < 24.99 kg/m^2^: normal weight; 25.0 kg/m^2^ ≤ BMI < to 29.99 kg/m^2^: overweight; and BMI ≥ 30.0 kg/m^2^: obese.

### 2.3. Lipid Profile

Because obesity is often related to dyslipidemia, the biochemical characterization of the patients included the determination of lipid profile. Total cholesterol, HDL, LDL, VLDL, and triglyceride were measured by specialized personnel from Hospital General Dr. Santiago Ramón y Cajal (ISSSTE) using Abbot Automated Clinical Biochemical Equipment, Spectrum Series II (Abbott Park, IL), and reagents of the same commercial brand.

### 2.4. Measurement of Leptin

Leptin concentrations in plasma were measured with the enzyme-linked immunosorbent assay, using the Human Leptin ELISA kit (Millipore; sensitivity 0.5 ng/mL). The absorbance was measured at 450/630 nm using the STAR FAX 303/PLUS (Awareness Technology, Inc.). Leptin concentrations were measured in triplicate and calculated from standard curves generated for each assay from the recombinant human leptin provided in the kit.

### 2.5. Statistical Analysis

The prevalence of obesity in Mexican men is 24.1% [14], which was adequate to calculate our sample size with a variation of up to 10%, as follows:

(1) $$\begin{array}{c} n=\frac{Z^{2}\;pqN^{2}}{E^{2}\left(N-1\right)}+1.96^{2}\;{pq}^{11}. \end{array}$$

See [15], where *n* = the sample size; *Z* = the standard normal deviation (1.64 for 90% confidence level); *E* = the level of sampling error (0.10); *p* = the proportion of the population that is overweight or obese; and *q* = the proportion of the population that is of normal weight.

Based on the population of Durango state, Mexico, we calculated a sample size of *n* = 49 for each group. Our total sample included 128 individuals.

For each parameter (age, blood pressure, BMI, leptin, cholesterol, HDL, LDL, and VLD), the collected data were organized in an Excel spreadsheet and then exported to the Graph pad Instat Version 7 software [16] to obtain the central tendency (means) and dispersion measures (standard deviation). Then, data were submitted to the Shapiro Wilk normality test to define the normality of each variable. After that, data corresponding to the four subgroups were compared using either a parametric (Student *t*-test) or a nonparametric test (Mann-Whitney), when distribution was considered normal and not normal, respectively. Finally, the distribution, mean, standard deviation, median, and minimum and maximum values of each parameter in both Tepehuán and Mestizo groups were compared according to their nutritional status, using a parametric (one-way ANOVA or Kruskal-Wallis) or nonparametric (Tukey-Kramer) test. On the other hand, the Pearson analysis was used to evaluate the correlation between leptin levels and BMI and biochemical parameters, in both Tepehuán and Mestizo groups, according to nutritional status. Values of *P* ≤ 0.05 were considered statistically significant.

## 3. Results

We studied a set of 128 clinically healthy male volunteers from Durango, Mexico, aged 18 to 59 years of age. Of these, 62 (30 Mestizo and 32 Tepehuán subjects) were of normal weight (NW) and 66 (38 Mestizo and 28 indigenous Tepehuán) were overweight or obese (OW/O; BMI > 25 kg/m^2^). The following parameters were recorded for each subject: demographic data, lipid profile, and leptin levels in plasma (Table 1).

The OW/O Tepehuán subjects presented the highest median age (39 years), in contrast to the NW Mestizo (21 years), NW Tepehuán (32.5 years), and OW/O Mestizo subjects (25 years); however, no statistically significant differences were observed among the four groups according the Student *t*-test. Moreover, the Kruskal-Wallis test (nonparametric ANOVA) showed no difference between ethnicities of the same nutritional status (*P* > 0.05). Independent of their nutritional status (as estimated by BMI), the 128 subjects presented no pathologic values for all biochemical parameters, including blood pressure, total cholesterol, HDL, LDL, VLDL, and triglycerides. These data suggest that the subjects are clinically healthy, although some are overweight or obese.

Mean blood pressure (MBP) was similar among all groups. No significant differences were observed in height or weight (*P* > 0.05). As expected, differences in BMI were evident among groups of different nutritional status independent of ethnicity. Interestingly, BMI was significantly lower in OW/O Tepehuán subjects (27.7 kg/m^2^) compared with OW/O Mestizo subjects (28.9 kg/m^2^) (*P* < 0.001).

**Figure 1:**
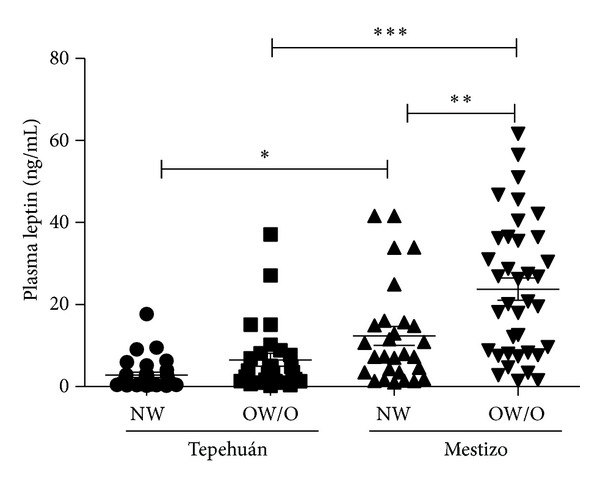
Comparison of circulating leptin levels in Tepehuán and Mestizo subjects according to their nutritional status. Distribution of the data is significantly different among the four subgroups according to ANOVA test (*P* < 0.0001). Moreover, analysis of the data by the Tukey-Kramer multiple comparisons test evidenced significant differences between NW Tepehuán and NW Mestizo, OW/O Tepehuán and OW/O Mestizo, and NW Mestizo and OW/O Mestizo subjects. *P < 0.05; **P < 0.01; ***P < 0.001. NW: normal weight; OW/O: overweight/obese.

Cholesterol values were similar among groups: NW Tepehuán, 146.5 mg/dL (range 107 to 188); NW Mestizo, 180 mg/dL (range 121 to 223); OW/O Tepehuán, 155.5 mg/dL (range 96 to 232) and OW/O Mestizo, 165 mg/dL (range 70 to 215). Median values for HDL and LDL were very similar among groups, with a wider range of HDL values in the OW/O Mestizo population (6–71 mg/dL) and a wider range of LDL values in OW/O Tepehuán group (37–139 mg/dL). The Mestizo population had a higher percentage of individuals with abnormal cholesterol levels (12%), and the OW/O Tepehuán population had abnormal levels of triglycerides (70%). Leptin concentrations varied considerably in each group (Figure 1). In the Mestizo population, median values were significantly higher in OW/O individuals (median: 20.45 ng/mL; range: 1.4 to 61.6 ng/mL) than in NW subjects (median: 7.66; range: 1.04 ng/mL to 41.6 ng/mL; *P* = 0.0025, IC_95_ = 4.1 to 18.3). Similarly, plasma leptin concentrations were significantly higher in the OW/O indigenous population (median, 6.5 ng/mL; range: 0.18 to 37.1 ng/mL) than in NW Tepehuán subjects (median: 2.9 ng/mL; range: 0.2 to 17.6 ng/mL; *P* = 0.03; CI_95_ 0.3 to 6.8); however, leptin levels were lower in Tepehuán than in Mestizo subjects, independent of BMI.

Pearson analysis demonstrated an association between circulating leptin and several biochemical parameters and BMI (Table 2). Interestingly, these correlations are different between Tepehuán and Mestizo subjects with the same nutritional status. In NW groups, leptin was negatively correlated with BMI in Tepehuán subjects but not in Mestizo subjects (*r* = −0.05; *P* = 0.05). In contrast, it was positively correlated with HDL in Tepehuán subjects but not in Mestizo subjects (*r* = 0.73; *P* = 0.02). In OW/O groups, leptin was positively associated with triglycerides (*r* = 0.70; *P* = 0.04) and LDL (*r* = 0.70; *P* = 0.04) in Mestizo subjects but not in Tepehuán subjects (*r* = 0.5, *P* = 0.37, and *r* = 0.5, *P* = 0.36, resp.). No significant associations were observed between leptin concentrations and the other parameters.

## 4. Discussion

Differences in the adipose-tissue secretory profile, as measured by adipokine levels, may play a role in racial/ethnic disparities in cardiovascular disease (CVD) [17].

Adipose tissue is an endocrine organ that produces many cytokines and hormones (adipokines) in a regulated manner. Leptin is one of these adipokines and has a variety of physiological roles related to the control of metabolism and energy homeostasis, acting on the central and peripheral systems. Leptin is closely linked to obesity and its complications, especially metabolic syndrome, diabetes mellitus, and CVD [18]. Moreover, several studies have showed great heterogeneity in leptin levels according to ethnicity, but this is the first such report on the indigenous Tepehuán population.

As it has been reported in other populations [19] we confirmed here that circulating leptin levels were higher in overweight/obese subjects from both ethnicities compared with normal weight individuals; however, Tepehuán subjects exhibited lower leptin concentrations than Mestizo subjects, independent of their BMI. Moreover, leptin concentrations remained in the normal range, even in overweight/obese indigenous subjects.

As expected, OW/O Mestizo individuals showed increased leptin levels, indicating that they present leptin resistance. The fact that OW/O Tepehuán present relatively low leptin levels suggests that obesity is not under by the same biochemical parameters in both populations could be related to differences in the nutritional qualities of the diet, which could affect fatness degree and therefore leptin levels. Another possibility is that the BMI cut-off value of 25 kg/m^2^ is not an appropriate parameter to define the nutritional status of Tepehuán indigenous subjects, in agreement with works showing that associations between BMI, body fat percentage, and distribution differ across populations, probably due to differences in body proportions and genetic background [20]. In future studies, it would be desirable to consider body fat percentage and water/muscle proportion, in addition to BMI, in order to better define the nutritional status of each individual. Unfortunately, this was not possible in the present study.

Our results are in agreement with those of Liuzzi et al. [21], who reported that leptin concentrations were higher in obese subjects than in normal subjects, with no differences between diabetics and nondiabetics. Leptin concentrations were not associated with age and showed a strong negative association with energy consumption only in women with a normal BMI. Leptin concentrations were directly correlated with BMI and body fat (expressed as percentage of total body mass and total fat mass), regardless of age and sex. Weight gain resulting from genetic, environmental, and nutritional factors plays an important role in the development of various metabolic disorders [22]. This weight gain is usually correlated with an increase in leptin levels, leading to leptin resistance in the long term. Leptin has also been associated with type-2 diabetes mellitus and the insulin resistance characteristic of this disease [23]. Poveda et al. [24] reported that the overall leptin concentration in 5-year-old children was 8.3 ± 8.9 ng/mL. Concentrations varied with age, sex, and body composition, with higher values in children with high BMIs and body fat percentages. Leptin concentrations were elevated in girls with increased levels of cholesterol and triglycerides, suggesting that changes in leptin are related to age and gender and to hormonal changes and differences in style of feeding. The variation in mean leptin values across ethnicities may help explain ethnic differences in the Tepehuán population in order to detect insulin resistance and hypertension risk because higher leptin is associated with hypertension independent of risk factors, anthropometric measures, and other ethnicities [25].

Viso-González et al. [26] evaluated 166 children and young Venezuelans (91 of normal weight and 75 obese, aged 2–15 years) of low socioeconomic status to determine serum leptin levels. Leptin levels were significantly higher in obese than in normal weight individuals, with no differences by gender or age. The lower bound of the 90th percentile for leptin concentration was 11.53 *μ*g/L in healthy children and 24.29 *μ*g/L in obese children. There was an inverse correlation between serum leptin and fat intake and waist/thigh index (RCM); excessive consumption of fats was associated with decreased serum leptin. These results suggest that obese children are resistant to leptin, regardless of age or gender. Misra et al. [27] investigated the relationships between plasma leptin concentration and obesity and diabetes and hyperlipidemia in individuals from India who were predisposed to abdominal obesity and metabolic syndrome. The results of that study suggest that plasma leptin has a strong positive correlation with total body fat percentage in that population. Among the components of metabolic syndrome, abdominal obesity is only weakly correlated with serum leptin levels. Tepehuán subjects of normal weight showed a negative correlation between leptin levels and BMI and media to leptin levels 1.9 ng/mL, which were the same correlations in the healthy children.

Our results indicated that physiological concentrations of leptin (2–5 ng/dL) might be related to triglyceride levels in overweight/obese Mestizo subjects but not in overweight/obese Tepehuán individuals. In Tepehuán subjects of normal weight with leptin levels below 5.0 ng/mL, the correlation with HDL was positive (*r* = 0.73,  *P* = 0.02), while in overweight/obese Mestizo subjects, leptin concentration was positively correlated with triglyceride (*r* = 0.70,  *P* = 0.04) and LDL (*r* = 0.70,  *P* = 0.04) levels.

Obesity is related to an increase in the number and size of adipocytes, which results in changes in adipokines synthesis, including leptin. Indeed, obese patients have higher levels of leptin and present leptin resistance. Several works have shown that both central and peripheral leptin resistance may be associated with different pathophysiological conditions related to obesity, such as inflammation, insulin resistance, and cardiovascular diseases (hypertension, atherosclerosis, obesity, ischemic heart disease, and heart failure), as well as hyperlipidemia [28, 29]. The high correlation between leptin levels and triglycerides or LDL content evidenced in the OW/O Mestizo subjects but not in the OW/O Tepehuán group could suggest a protection status in indigenous group, when compared with the mestizo population. We can speculate that the relatively low leptin concentration in OW/O Tepehuán could protect them against the development of atherosclerosis. Hyperleptinemia is usually associated with a progressive increase in body weight and the development of metabolic syndrome, leading to global or selective leptin resistance. Both central and peripheral leptin resistance may be present under pathophysiological conditions such as inflammation, insulin resistance, hyperlipidemia, and other CVDs, including hypertension, atherosclerosis, obesity, ischemic heart disease, and heart failure [30, 31].

Unfortunately, our results cannot be extrapolated to the entire indigenous or Mestizo Mexican population, because there are around 64 indigenous groups in Mexico, who live in different environmental, dietary, and geographic conditions; moreover, they all have their own genetic background. Even though individuals share 99.9% of their genome, there are individual genomic differences that make them all different. Thus studies carried out by our group showed ethnic differences in genetic polymorphisms of genes that regulate CYP450 enzymes among Amerindian of Tepehuán origin, Menonitas, and Mestizo populations living in Durango State, Mexico [20, 32]. In addition, the sample size was determined for the population of the Durango state and not for the total population of Mexico. This point is now discussed in the revised version of our paper. Additional studies involving a larger number of participants, including other indigenous groups and Mestizos Mexican from other regions of Mexico, should be performed to be able to extrapolate the data to the Mexican population as a whole.

## Figures and Tables

**Table 1:** Comparative analysis of biochemical parameters of Tepehuán and Mestizo subjects from Durango, Mexico, according to their nutritional status (BMI).

Variable	Normal value	Normal weight (*n* = 62)			Overweight/obese (*n* = 66)		
		Tepehuán (*n* = 32)	Mestizo (*n* = 30)	*P *	Tepehuán (*n* = 28)	Mestizo (*n* = 38)	*P *
Leptin (ng/mL)	3–18	1.9 (0.23 to 17.6)	7.66 (1.04 to 41.6)	<0.001^ab^	3.6 (0.18 to 37.1)	20.1 (1.4 to 61.65)	<0.001^ab^
Triglycerides (mg/dL)	10–150	103 (44 to 243)	185 (60 to 279)	<0.05^ab^	190 (62 to 525)	103 (42 to 464)	<0.05^a^
VLDL (mg/dL)	2–30	20.5 (9 to 49)	37 (11 to 56)	>0.05^a^	38.5 (12 to 97)	19.5 (8 to 93)	<0.001^ab^

Data are shown as mean values with minimum and maximum values in parentheses.

BMI: body mass index; NW: normal weight; OW/O: overweight/obese; VLDL: very low density lipoproteins.

^a^Tepehuao versus Mestizo using Kruskal-Wallis (nonparametric ANOVA) comparison test and Mann-Whitney *posthoc* test.

^b^Tepehan versus Mestizo using Tukey-Kramer comparison test.

**Table 2:** Correlation of leptin concentration with BMI and biochemical parameters, according to nutritional status (BMI).

Variable	Normal weight (*n* = 62)				Obese/overweight (*n* = 66)			
	Tepehuán (*n* = 32)	*P* value	Mestizo (*n* = 30)	*P* value	Tepehuán (*n* = 28)	*P* value	Mestizo (*n* = 38)	*P* value
BMI	−0.5 (−1.0 to 2.7)	0.05	0.06 (−1.55 to 2–08)	0.40	−0.06 (−3.37 to 0–20)	0.20	0.02 (−1.21 to 0–81)	0.84
Cholesterol	0.62 (−0.09 to 0.04)	0.12	0.48 (−0.07 to 0.05)	0.40	0.40 (−0.10 to 0.06)	0.63	0.28 (−0.01 to 0.23)	0.88
Triglycerides	0.38 (−0.003 to 0.02)	0.67	0.38 (−0.03 to 0–05)	0.68	0.50 (−0.02 to 0.02)	0.37	0.70 (−0.02 to 0.11)	0.04
HDL	0.73 (−0.17 to 0.24)	0.02	0.30 (−0.37 to 0.15)	0.84	0.60 (−0.43 to 0.53)	0.16	0.56 (−0.20 to 0.33)	0.23
LDL	0.43 (−0.14 to 0.08)	0.54	0.40 (−0.12 to 0.07)	0.62	0.5 (−0.18 to 0.09)	0.36	0.70 (−0.07 to 0.27)	0.04
VLDL	0.36 (−0.14 to 0.08)	0.7	0.36 (−0.12 to 0.07)	0.7	0.39(−0.18 to 0.09)	0.66	0.36 (−0.07 to 0.27)	0.72

Data are shown as mean values with minimum and maximum values in parentheses.

BMI: body mass index; NW: normal weight; OW/O: overweight/obese; HDL: high density lipoproteins; LDL: low density lipoproteins; VLDL: very low density lipoproteins.
